# Effect of chest compression with kneeling on the bed in clinical situations

**DOI:** 10.1111/jjns.12314

**Published:** 2020-01-19

**Authors:** Tomoyuki Hasegawa, Ritsu Okane, Yoko Ichikawa, Sayuri Inukai, Shin Saito

**Affiliations:** ^1^ Mie Prefectural College of Nursing Tsu Japan

**Keywords:** cardiopulmonary resuscitation, chest compression, fatigue, grounding toe, rescuer's position

## Abstract

**Aim:**

Cardiopulmonary resuscitation is vital for survival after cardiac arrest, and chest compressions are an important aspect of this. When performing chest compression in a hospital setting, the rescuer often has to kneel on the bed to overcome inconvenient differences in height between the rescuer and the bed. However, as yet no study has evaluated the quality of chest compressions in this position. The aim of this study was to examine the impact on the quality of chest compressions while kneeling on the bed.

**Methods:**

Fifteen female students performed 2‐min chest compressions on a manikin placed on the floor and a bed. Measurement parameters included compression depth, heart rate, integrated electromyogram, and a visual analog scale. The parameters were measured every 30 s and were statistically compared between the conditions.

**Results:**

Compression depth at 30, 60, 90, and 120 s differed significantly between the conditions. Heart rate values at 150 and 210 s of recovery significantly differed between the conditions. Integrated electromyogram values for the trapezius, rectus femoris, and biceps femoris differed between the floor and bed conditions during 2‐min chest compressions, whereas the external oblique muscle significantly differed at 60 and 120 s. Visual analog scales for fatigue, effectiveness, and stability significantly differed between the conditions.

**Conclusion:**

Kneeling on the bed does not enable grounding of the toe, causing the upper body to be unstable and limiting generation of the power required for chest compression. Our results suggest that rotation every minute is necessary to maintain effective cardiopulmonary resuscitation while kneeling on the bed.

## 1 INTRODUCTION

High‐quality cardiopulmonary resuscitation (CPR) is vital for survival after cardiac arrest, and chest compressions are an important aspect of this (Abella et al., 2005a; Berg et al., 2010; Perkins et al., 2015). Providing early and effective chest compression in these patients improves the chances of survival and of neurologically favorable outcomes (Heidenreich, Bonner, & Sanders, 2012; Holmberg, Holmberg, & Herlitz, 2000; Nagao, 2009; SOS‐KANTO study group, 2007). All hospital staff members should therefore be able to perform chest compressions of the highest possible quality (Heng, Fong, Wee, & Anantharaman, 2011).

To maximize the effectiveness of chest compressions, the patient should if possible be placed in a supine position on a firm surface, with the rescuer either kneeling beside the patient's chest (when the patient is on the ground) or standing beside the bed (e.g., in a hospital setting) (Berg et al.; Handley & Handley, 2004). The “kneeling posture” is recommended for chest compression in out‐of‐hospital situations; this requires the rescuer to kneel as close to the patient as possible, place his or her body directly above the patient's chest, straighten both arms, and place the heel of the palm on the lower half of the sternum with the fingers interlocked (Perkins et al.).

Most CPR procedures in hospitals are performed with the patient lying on a medical bed (Berg et al.). The most effective bed height position is where the patient's chest is level with the rescuer's mid‐thigh, which allows the rescuer to achieve the greatest intrathoracic pressures during CPR (Lewinsohn, Sherren, & Wijayatilake, 2012). It is difficult to adjust the bed height according to the height of the individual rescuer each time resuscitation is required, and high fixed‐height beds affect the quality of chest compressions provided by individuals of a shorter stature (Lee, Kim, Kim, & Lee, 2012). The effectiveness of the chest compressions can therefore differ according to the relative heights of the rescuer and bed.

The rescuer may therefore need to kneel on the bed while performing chest compressions. It is presumed that this position offsets the inefficiency arising from the mismatch in height between the bed and rescuer. A kneeling posture with a kneeling stool was preferred by participants, which have shown similar results in chest compression parameters and visual analog scales (VAS) with a standing posture on a stepstool with bed height adjustment during in‐hospital CPR (Oh, Chee, Lim, Cho, & Kim, 2014). However, the kneeling stool is not commonly used. To the best of our knowledge, there have been no studies that compared the quality of chest compressions in kneeling on the bed and floor position. The aim of the present study was therefore to investigate the impact of kneeling on the bed on the quality of chest compressions.

## 2 METHODS

### 2.1 Participants

The present study included 15 female students with no history of any musculoskeletal or functional mobility issues who had undergone recent training in basic life support (BLS) at the college. After participants provided written informed consent, data regarding age, height, weight, body mass index (BMI), and exercise habits were obtained. Sample size adequacy (n = 15) using t test, G power 3.1.9.4 analysis software was estimated based on alpha level of 0.05, effect size of 0.90, and power of 0.95. The sample size of this study was appropriate.

### 2.2 Protocol

**Figure 1:**
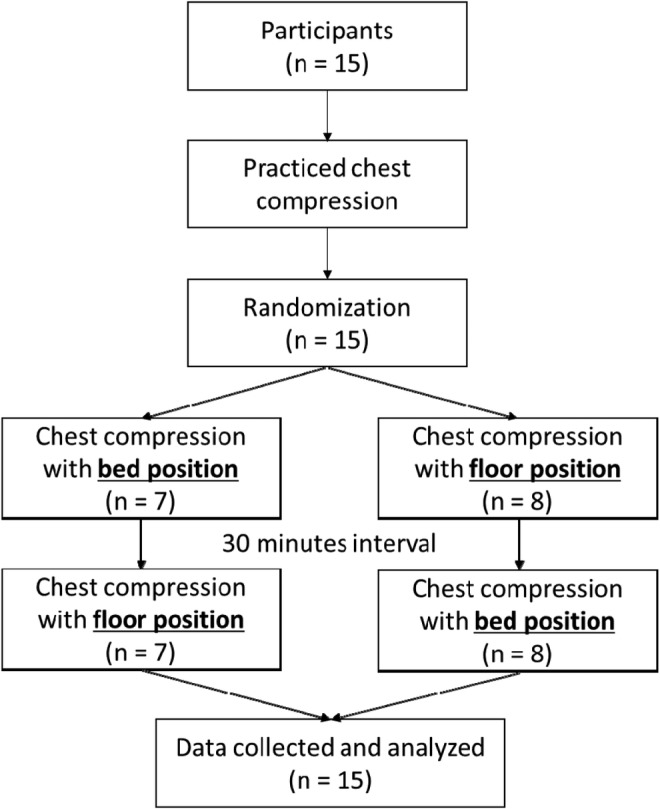
Experimental protocol

The study protocol is summarized in Figure 1. Prior to the experiment, the participants underwent sufficient training and practiced chest compressions until they could consistently perform at least 5 cm of compression and maintain a chest compression rate of at least 100/min. A researcher checked that they were able to perform chest compressions effectively equivalent to a BLS healthcare provider.

**Figure 2:**
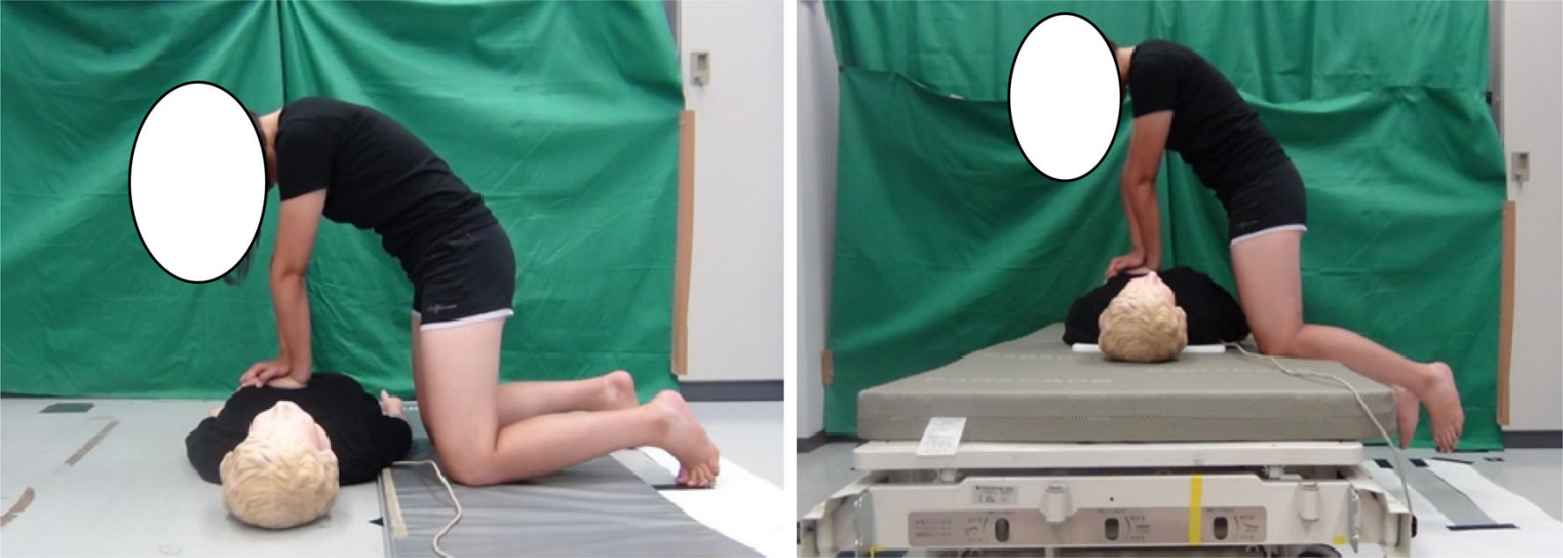
Positions for the chest compressions on the floor (left) and bed (right)

During the experiment, each participant performed chest compressions in two different surface conditions (i.e., on the floor and on a bed, in a randomized order) separated by a 30‐min interval. Each task included a 5‐min rest followed by chest compressions for 2 min and recovery for 2 min. During the 5‐min rest period, participants sat with their eyes closed while wearing electromyography (EMG) and electrocardiography (ECG) electrodes. This period was followed by 2 min of chest compressions performed without any audiovisual feedback on a Resusci Anne SkillReporter manikin (Leardal Medical Corporation, Stavanger, Norway) that had been placed on the floor or on a bed (Figure 2). In the floor condition, the manikin was on the floor and the participant kneeled on a thin mattress. In the bed condition, the manikin was placed on a mattress (910 × 1910 × 85 mm, KE‐601, Paramount Bed Co., Ltd, Tokyo, Japan) with a backboard. A compression rate of 100/min was maintained using a metronome (Park, Hong, Shin, Lee, & Hwang, 2013). In both conditions, the participant kneeled beside the manikin with a distance between their knees of 400 mm, and a distance between the center of the manikin and participant's knees of 250 mm. After completing the chest compressions, participants sat and recovered in a comfortable posture.

### 2.3 Data collection

Compression depth (CD) was recorded as each participant performed chest compressions on the manikin. Heart rate (HR) was measured at rest, during chest compression, and during recovery using a Life Scope 8 (Nihon Kohden Co., Tokyo, Japan). During chest compressions, the surface EMG collected data from the triceps brachii, deltoid, trapezius, erector spinae, external oblique muscle, rectus femoris, biceps femoris, and medial gastrocnemius muscles using SX230‐1000 electrodes (Biometrics Ltd., UK) (Trowbridge et al., 2009; Yasuda et al., 2013). The electrodes were attached to the belly of each muscle, and the skin was abraded and cleaned with alcohol before attachment to minimize impedance. Analog HR and EMG signals were sampled at 1.5 kHz using an AD16‐16 (PCI) EV A/D converter (Contec Co. Ltd., Osaka, Japan), and stored in a personal computer using a G1 system Analog Recorder Pro ver. 1.60 (G1 system, Aichi, Japan). Between the chest compressions and recovery, participants were asked to provide their subjective evaluation 10 items (their fatigue, effectiveness, and sense of stability, and the loads experienced by their shoulders, arms, back, belly, waist, thighs, and calves) using a 100‐mm VAS, with higher scores representing greater fatigue, effectiveness, stability, and loads. “Effectiveness” was the participant's assessment of how effectively they felt the chest compression had been performed; and “sense of stability” was the participant's assessment of how stable the trunk was during chest compression.

### 2.4 Evaluation

The quality of chest compressions was assessed using the median CD per 30‐s period (McDonald, Heggie, Jones, Thorne, & Hulme, 2013). HR was evaluated during chest compressions and recovery as changes from the initial baseline value after resting. Surface EMG signals were full‐wave rectified and integrated over 30 s during chest compressions to determine muscle activity **(**Yasuda et al.). Fatigue, effectiveness, sense of stability, and load in each part after 2 min of chest compressions were determined by matching against a VAS.

### 2.5 Statistical analysis

The compressions performed on the floor and on the bed were statistically compared. The all data were compared using the Wilcoxon signed rank test. Data were statistically analyzed using SPSS/PASW Statistics ver. 18.0 (IBM, Armonk, NY, USA), and the significance level was set at .05.

### 2.6 Ethics approval and consent to participate

The research was approved by the author's university ethics review board. (Approval No. 120203). Experimental protocols and procedures were explained to all those who responded to public advertisements regarding the study, and all participants voluntarily provided written informed consent. High priority was given to the safety of the participants, and appropriate measures were taken to allow and process the withdrawal of any participant from the study at any time.

## 3 RESULTS

### 3.1 Participants

The mean age of the participants was 21.1 ± 0.3 years, and the mean height, body weight, and BMI were 160.4 ± 5.8 cm, 50.7 ± 6.8 kg, and 19.7 ± 2.2 kg/m^2^, respectively. These values were almost equivalent to the typical Japanese body characteristics for women (Research Institute of Human Engineering for Quality Life, 2011). Exercise habits in all participants were observed.

### 3.2 Compression depth

**Figure 3:**
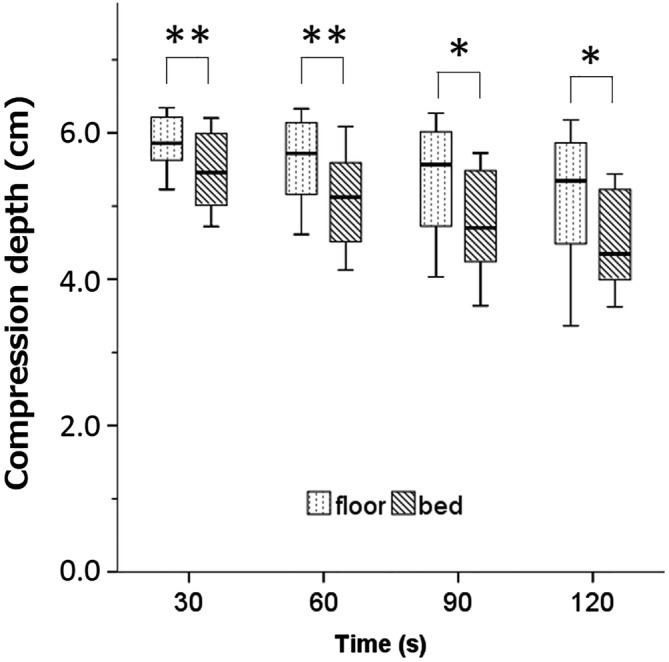
Comparison of compression depth between the floor and bed conditions. Values are shown as medians and ranges using a boxplot. Significant differences were observed between the conditions (*p < .05 and **p < .01; Wilcoxon signed rank test).; n = 15

The median CD over 30‐s periods in the floor and bed conditions ranged from 5.86 to 5.34 cm and from 5.46 to 4.35 cm, respectively (Figure 3). Compressions performed in the floor condition did not decline over the 2 min; however, median CD decreased to <5 cm after 60 s in the bed condition. Values differed significantly between the floor and bed conditions at 30 s (5.86 vs. 5.45 cm, *p* < .01), 60 s (5.72 vs. 5.12 cm, *p* < .01), 90 s (5.57 vs. 4.70 cm, *p* < .05), and 120 s (5.34 vs. 4.35 cm, *p* < .05).

### 3.3 Heart rate

**Figure 4:**
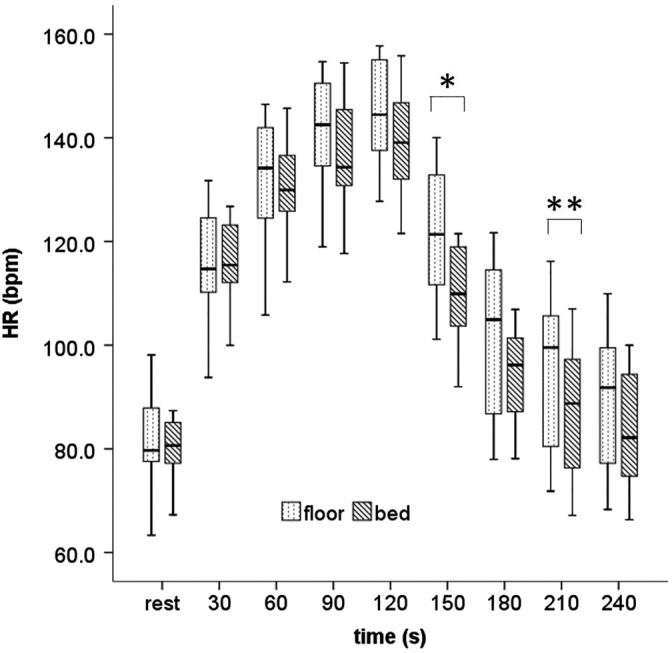
Comparison of heart rate between the floor and bed conditions. Values are shown as medians and ranges using a boxplot. Significant differences were observed (*p < .05 and **p < .01; Wilcoxon signed rank test). HR, heart rate. The chest compression phase was between rest and 120 s, and the recovery phase was between 120 and 240 s.; n = 15

Figure 4 shows the HR during the chest compression and recovery periods. The median and interquartile range values of HR increased at the end of 120 s of compressions to 144.5 (136.7–155.3) and 139.1 (130.5–148.2) bpm in the floor and bed conditions, respectively, and had declined 120 s later (at the end of the recovery period) to 91.8 (76.0–99.6) and 88.2 (74.7–94.8) bpm in the floor and bed conditions, respectively. These values differed significantly between the conditions at 150 s (121.4 vs. 109.9 bpm, *p* < .05) and 210 s (99.5 vs. 88.8 bpm, *p* < .01) during the recovery period.

### 3.4 Muscle activity

**Figure 5:**
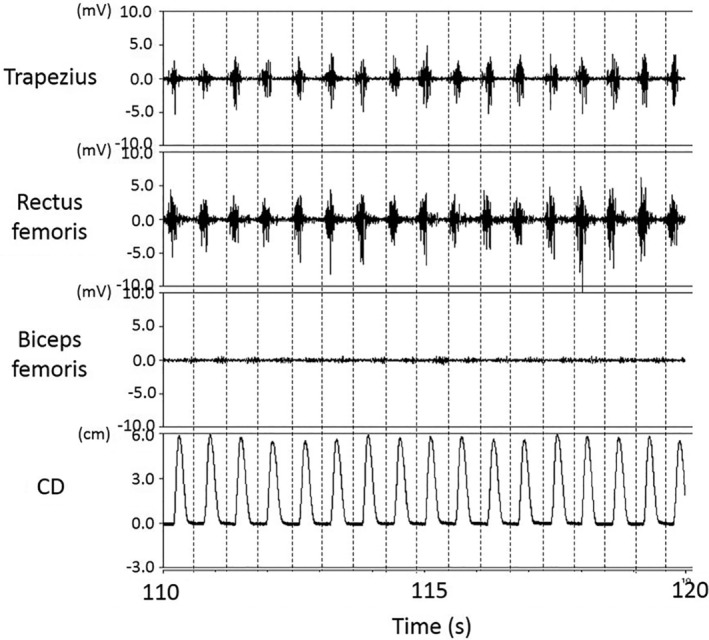
Electromyogram characteristics of one participant during the final 10 s of chest compressions in the floor condition. CD: compression depth. The dashed line shows the moment of chest compression

**Figure 6:**
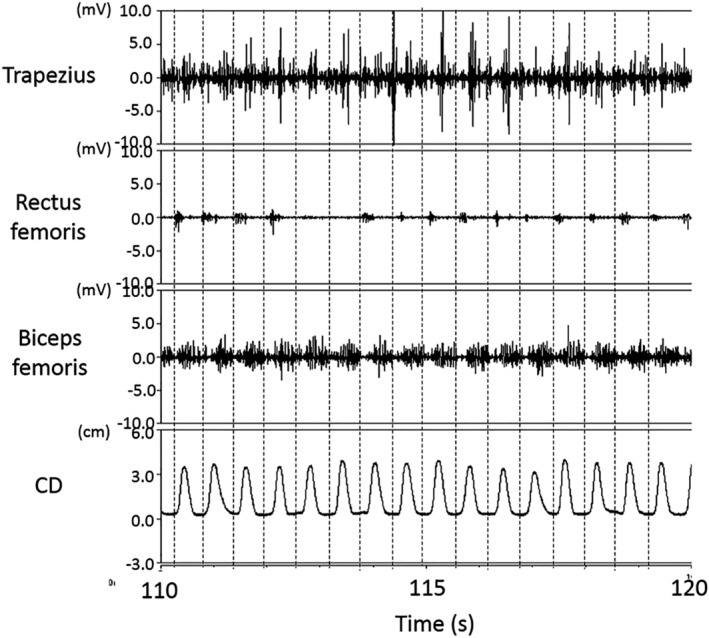
Electromyogram characteristics of one participant during the final 10 s of chest compressions in the bed condition. CD: compression depth. The dashed line shows the moment of chest compression

The median integrated EMG values differed significantly between the floor and bed conditions for each 30‐s period during the chest compressions for the trapezius (30 s; 7.8 vs. 12.0 mV, *p* < .01, 60 s; 7.8 vs. 11.3 mV, *p* < .01, 90 s; 8.0 vs. 10.7 mV, *p* < .01, 120 s; 7.5 vs. 9.3 mV, *p* < .01, respectively), rectus femoris (30 s; 6.2 vs. 2.5 mV, *p* < .01, 60 s; 6.5 vs. 2.7 mV, *p* < .01, 90 s; 7.2 vs. 2.2 mV, *p* < .01, 120 s; 6.0 vs. 2.5 mV, *p* < .01, respectively), and biceps femoris (30 s; 5.0 vs. 7.7 mV, *p* < .01, 60 s; 5.3 vs. 8.9 mV, *p* < .01, 90 s; 4.4 vs. 9.7 mV, *p* < .01, 120 s; 3.9 vs. 8.4 mV, *p* < .05, respectively); the external oblique muscle values differed significantly at 60 and 120 s (60 s; 6.5 vs. 6.0 mV, *p* < .05, 120 s; 5.4 vs. 5.8 mV, *p* < .05, respectively); and the triceps brachii, deltoid erector spinae, and medial gastrocnemius muscle did not exhibit any significant differences between the conditions (Table 1). Figures 5 and 6 show EMG characteristics during the final 10 s of chest compressions for one participant (the same participant in both cases). Muscle activity was periodically and immediately observed before compression in the floor condition (Figure 5). However, in the bed condition this participant exhibited remarkable biceps femoris muscle activity during decompression, rectus femoris activity was not seen, and the trapezius activity was irregular (Figure 6). All the other participants showed the same trend.

**Table 1:** Comparison of integrated electromyogram values between the floor and bed conditions

	30	60	90	120 (s)
Triceps brachii (mV)				
Floor	20.1 (16.2–24.5)	21.1 (15.8–26.0)	21.4 (14.1–26.4)	20.2 (13.8–25.5)
Bed	20.7 (15.8–31.7)	22.2 (17.8–30.1)	22.3 (18.7–28.8)	22.0 (19.0–24.9)
Deltoid (mV)				
Floor	21.1 (12.3–23.8)	22.1 (13.5–23.3)	19.2 (11.2–23.5)	18.8 (10.4–23.9)
Bed	21.2 (9.8–34.0)	19.7 (10.4–28.6)	18.4 (11.3–30.2)	15.9 (10.1–27.9)
Trapezius (mV)				
Floor	7.8 (6.4–10.4)**	7.8 (6.5–9.9)**	8.0 (6.0–10.0)**	7.5 (5.7–9.8)**
Bed	12.0 (9.1–13.9)	11.3 (9.0–12.7)	10.7 (9.6–12.8)	9.3 (8.1–12.1)
Erector spinae (mV)				
Floor	4.7 (4.0–6.8)	4.8 (4.3–7.8)	4.9 (4.3–7.4)	4.8 (4.0–6.9)
Bed	4.6 (4.2–6.4)	5.0 (4.4–8.1)	5.0 (4.5–7.8)	4.8 (4.1–8.1)
External oblique (mV)				
Floor	6.1 (3.7–11.5)	6.5 (3.9–12.2)*	6.0 (3.9–12.9)	5.4 (3.9–12.0)*
Bed	5.0 (3.0–8.2)	6.0 (3.4–8.1)	5.6 (3.5–8.1)	5.8 (3.2–7.8)
Rectus femoris (mV)				
Floor	6.2 (4.8–9.8)**	6.5 (4.8–14.1)**	7.2 (5.0–11.9)**	6.0 (5.4–10.0)**
Bed	2.5 (1.5–3.2)	2.7 (1.7–3.5)	2.2 (1.7–3.2)	2.5 (1.5–2.9)
Biceps femoris (mV)				
Floor	5.0 (3.8–6.3)**	5.3 (3.5–7.6)**	4.4 (3.4–7.2)**	3.9 (3.4–7.4)*
Bed	7.7 (4.9–11.2)	8.9 (5.1–10.4)	9.7 (5.8–10.9)	8.4 (5.0–11.0)
Medial gastrocnemius (mV)				
Floor	1.8 (1.6–3.0)	2.0 (1.7–3.4)	2.1(1.6–2.6)	1.6 (1.4–2.2)
Bed	1.8 (1.3–3.3)	2.3 (1.4–5.3)	2.3 (1.7–4.1)	1.9 (1.4–4.7)

Note: Values are median (interquartile range) over 30‐s periods. Significant differences between floor and bed conditions were observed. (*p < .05, **p < .01; Wilcoxon signed rank test); n = 15.

### 3.5 Visual analog scale

**Figure 7:**
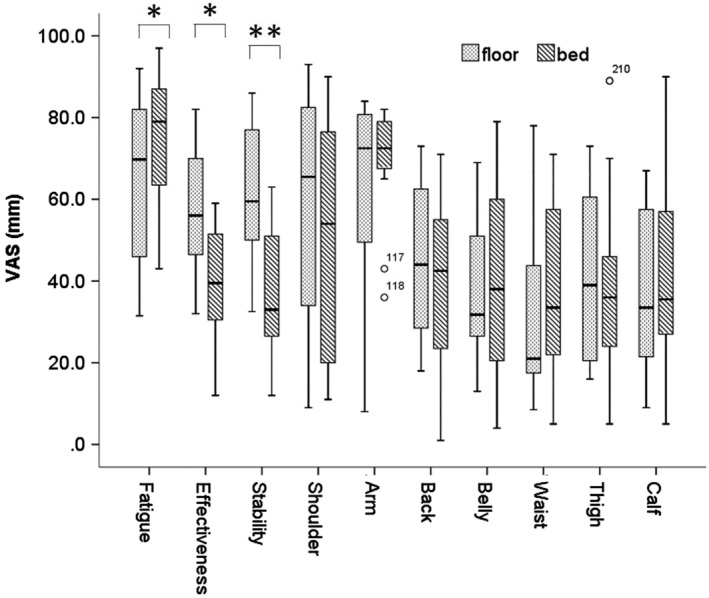
Comparison of visual analog scale values between the floor and bed conditions. Values are shown as medians and ranges using a boxplot. Significant differences were observed (*p < .05 and **p < .01; Wilcoxon signed rank test). VAS: visual analog scale. Each bar represents the participants' subjective assessment of their fatigue, effectiveness, and sense of stability, and of the load in their shoulders, arms, back, belly, waist, thighs, and calves.; n = 15

The median VAS values for fatigue (69.8 vs. 79.0 mm, *p* < .05), effectiveness (56.0 vs. 39.5 mm, *p* < .05), and sense of stability (59.5 vs. 33.0 mm, *p* < .01) differed significantly between the two conditions (Figure 7). However, the mean values for the load perceptions in each part of the body did not significantly differ between the conditions.

## 4 DISCUSSION

The 2015 American Heart Association guidelines for CPR and emergency cardiovascular care suggest that all rescuers, regardless of training, should provide chest compressions to victims of cardiac arrest (Kleinman et al., 2015). However, uninterrupted chest compressions reduce the number of adequate deep chest compressions the rescuer can provide and lead to physical fatigue (Bjorshol, Sunde, Myklebust, Assmus, & Soreide, 2011; Kleinman et al.; Shin et al., 2014). To be effective, chest compressions should be performed fast and hard. It is reasonable for laypersons and healthcare providers to compress the adult chest at a rate of 100–120 compressions per minute with a CD of 5–6 cm (Kleinman et al.). The present study confirmed that the quality of chest compressions provided in the bed condition changed in a similar manner to that observed in previous studies (Bjørshol et al., Shin et al.), and the CD declined to <5 cm after 60 s. Generally, the trunk is in a more horizontal position during kneeling. The downward push during chest compression can be achieved by using the weight of the trunk and eccentric contraction of the trunk extensors, whereas the upward lift of chest compression requires concentric contraction of the extensor muscles against the weight of the trunk (Jones & Lee, 2008). The rescuer performing chest compressions develops force by accelerating the upper body downward using gravity and uses hip extension torque to hold the trunk up against the inertial force of gravity during decompression (Jones, & Lee). The main difference between the two conditions was with regard to the grounding of the rescuer's toes. Instability during chest compression in the bed group was influenced by movement of the fulcrum of the knee and hip and the small size of the base of the support. In addition, the quality of chest compression was influenced by instability of the whole body due to sinking a little by kneeling on the mattress. The lower thigh moves with compression and the muscle activity of the biceps femoris, a flexion muscle, increases. Therefore, the upper body is unstable if the toe is not grounded and is unable to generate the power required for chest compression. The increase in HR was lower than on the floor, and instability is thought to have influenced the participants' subjective fatigue and to shallow chest compression than on the floor. The environment in a moving ambulance results in a lower percentage of chest compressions that achieve adequate depth, compared to chest compressions performed stationary on the ground (Stone & Thomas, 1995). Future study should consider the impact on chest compressions of sinking into the mattress while kneeling.

On the floor, the participants maintained adequate compressions for 2 min. In this position, the lower body remains stable and the base of the support is larger in area, enabling participants to use their body weight during compression. In addition, chest compressions, in particular, utilize the muscular power of the back and thighs. Rescuers with lighter body weight produce the required force using the trapezius, abdominal rectus, external oblique, and rectus femoris muscles (Hasegawa, Daikoku, Saito, & Saito, 2014). If these rescuers do not undertake regular exercise they can become fatigued while performing chest compressions, which gradually reduces the quality of the compression (Hasegawa et al.). Although the participants' weights were similar to the Japanese average, the participants were able to correctly perform 2 min of chest compressions. Good physical fitness and the height of the rescuer are positively correlated with the quality of chest compressions, irrespective of sex (Hansen et al., 2012; Russo et al., 2011). Therefore, the participants of this study were able to perform effective chest compressions because of their exercise habits. The increase in HR in the participants during the floor condition was higher because it was easier for the participants to maintain an appropriate posture on the floor than on the bed, thus they could push harder on the mannequin's chest.

Kneeling on the bed prevents rescuers from providing continuous, high‐quality chest compressions. According to the guidelines, the rotation time for chest compressions should be approximately 2 min (Perkins et al.; Kleinman et al.). The results of this study showed that CD was <5 cm after 1 min when kneeling on the bed; therefore, we recommend rotation every minute to maintain effective CPR.

### 4.1 Limitations of the study

Future studies should consider the impact on the chest compressions of sinking into the mattress while kneeling. In this study, only women participated. Regarding the relationship between CPR quality and gender, indicators of exertion during CPR are higher in women than in men, but these gender differences are because of BMI and differences in physical fitness conditions (Lopez et al., 2015). It is necessary to clarify the relationship between gender and kneeling on a mattress. Furthermore, the participants of this study were students; the findings should be verified with healthcare professionals. There has been no research in recent years to clarify the quality of CPR in real situations. The quality of multiple parameters of CPR was inconsistent and often did not meet published guideline recommendations, even when performed by well‐trained hospital staff during in‐hospital cardiac arrest (Abella et al., 2005b). It is necessary to clarify the relationship between patient outcome and posture of chest compressions. Metronome guidance improved compression rate but did not affect CD (Oh et al., 2008) or made them shallower (Park et al.). It is necessary to examine without using a metronome. Despite some limitations, kneeling on the bed helped to overcome difficulties arising from the relative difference in height between rescuer and bed. Future studies are needed that focus on the development of a bed that enables grounding of the toes while kneeling on it.

## 5 CONCLUSIONS

The aim of this study was to examine the quality of chest compressions provided in the kneeling position on the bed. The results showed that the toes were not grounded in this position, causing the upper body to be unstable and does not permit cooperation of body weight and muscle, limiting the generation of the required power during compressions. These results not only highlight the importance of grounding the toes during chest compression but also provide evidence for recommending rotation every minute to maintain effective CPR while kneeling on the bed.

## CONFLICT OF INTEREST

There are no conflicts of interest to disclose.

## AUTHORS' CONTRIBUTIONS

T.H. performed the experiments, analyzed the data and wrote the manuscript. R.O., Y. I, S.I., and S.S., coordinated the study and supervised the collection and analysis of data. All authors have read and approved the final manuscript.
